# Finding low-conductance sets with dense interactions (FLCD) for better protein complex prediction

**DOI:** 10.1186/s12918-017-0405-5

**Published:** 2017-03-14

**Authors:** Yijie Wang, Xiaoning Qian

**Affiliations:** Department of Electrical & Computer Engineering, Texas A and M University, MS 3128, TAMU, College Station, TX, USA

**Keywords:** Protein complex identification, Low conductance set, Dense subnetwork, Mixed integer programming

## Abstract

**Background:**

Intuitively, proteins in the same protein complexes should highly interact with each other but rarely interact with the other proteins in protein-protein interaction (PPI) networks. Surprisingly, many existing computational algorithms do not directly detect protein complexes based on both of these topological properties. Most of them, depending on mathematical definitions of either “modularity” or “conductance”, have their own limitations: Modularity has the inherent resolution problem ignoring small protein complexes; and conductance characterizes the separability of complexes but fails to capture the interaction density within complexes.

**Results:**

In this paper, we propose a two-step algorithm FLCD (**F**inding **L**ow-**C**onductance sets with **D**ense interactions) to predict overlapping protein complexes with the desired topological structure, which is densely connected inside and well separated from the rest of the networks. First, FLCD detects well-separated subnetworks based on approximating a potential low-conductance set through a personalized PageRank vector from a protein and then solving a mixed integer programming (MIP) problem to find the minimum-conductance set within the identified low-conductance set. At the second step, the densely connected parts in those subnetworks are discovered as the protein complexes by solving another MIP problem that aims to find the dense subnetwork in the minimum-conductance set.

**Conclusion:**

Experiments on four large-scale yeast PPI networks from different public databases demonstrate that the complexes predicted by FLCD have better correspondence with the yeast protein complex gold standards than other three state-of-the-art algorithms (ClusterONE, LinkComm, and SR-MCL). Additionally, results of FLCD show higher biological relevance with respect to Gene Ontology (GO) terms by GO enrichment analysis.

## Background

Recent developments of high-throughput profiling techniques, such as yeast two-hybrid (Y2H) and tandem affinity purification (TAP) with mass spectrometry (MS), allow scientists to generate large-scale protein-protein interaction (PPI) datasets for different species [1–5]. These interactome data have enabled us to discover biological insights from a systematic point of view through PPI networks, where nodes represent proteins and edges denote biological relationships (either physical binding or statistical association) between two proteins. In this paper, we focus on predicting protein complexes in derived PPI networks from high-throughput profiling.

Based on the inherent topological structures of protein complexes [6], prediction of protein complexes can be formulated as searching for subnetworks that are densely connected inside and well separated from the rest of the PPI networks. Many algorithms have been developed and applied for this purpose of detecting protein complexes.

These existing algorithms can be grouped into three categories. The first category includes the algorithms that mimic Markovian random walk on graphs, pioneered by MCL [7]. MCL does not have explicit mathematical definitions for the desired properties of subnetworks to detect as protein complexes. Similar to random walk, it iteratively implements “Expand” and “Inflation” operations to generate non-overlapping complexes. R-MCL [8] and SR-MCL [9] are improved versions of MCL. R-MCL penalizes the large complexes at each iteration in order to obtain more size-balanced complexes with a similar number of nodes within them. SR-MCL executes R-MCL many times to yield overlapping complexes. All those algorithms have shown good empirical performance, despite the mystery of parameter tuning and the lack of theoretic understanding of their working mechanisms.

Algorithms in the second category do not directly predict complexes according to the topological structure of subnetworks but resemble traditional clustering methods based on derived similarity measures between nodes or edges. For example, MCODE [1], CFinder [10], and RRW [11] grow complexes from single nodes by iteratively adding similar nodes in terms of different similarity criteria that help form local dense subnetworks. However, they only concentrate on the internal connectivity of the subnetworks and neglect the connectivity between the subnetworks and the rest of the networks. LinkComm [12] represents networks with edge graphs, whose nodes are interactions and edges reflect the similarity between interactions, and derives potential complexes by hierarchical clustering to partition the edge graphs.

Algorithms in the third category detect complexes based on explicit topological definitions of protein complexes. For example, modularity [13] and conductance [6, 14] are two widely used definitions. Algorithms based on modularity [15] aim to detect subnetworks that have higher than expected internal connections. And algorithms, such as ClusterONE [6], based on finding low-conductance sets, focus on the separability of the subnetworks, which can be quantified by the ratios between the external connections of subnetworks and the total number of interactions of the proteins within the subnetworks. However, these methods have their own limitations. Modularity-based methods have the inherent resolution problem [16], which leads to ignorance of small-size protein complexes. Algorithms based on conductance minimization [6, 17] consider the relationships between the internal connections and the external connections of subnetworks, but neglect the density of the interactions within the subnetworks.

In this paper, we propose a two-step algorithm FLCD (**F**inding **L**ow-**C**onductance sets with **D**ense interactions) to detect protein complexes that have dense interactions inside and sparse interactions outside in a given PPI network. FLCD explicitly takes care of both the internal and external connectivity of protein complexes in two steps. FLCD first identifies a low-conductance set around a protein, which is locally well separated from the rest of the network. Then a densely connected subnetwork within the low-conductance set is detected based on the definition of the edge density of a subnetwork proposed in [18]. We compare our FLCD with three state-of-the-art overlapping complex prediction algorithms, which are ClusterONE [6], LinkComm [12], and SR-MCL [9], respectively. Experimental results on four different yeast PPI networks from different publicly accessible databases demonstrate that our FLCD outperforms all competing algorithms for biological significance in terms of yeast protein complex gold standards and Gene Ontology (GO) term annotations [19].

## Results and discussion

We first introduce the implementation details of the algorithms that we take for comparison; the information of the PPI networks, the reference protein complex datasets as our gold standards, and the GO terms we use for evaluation; and the criteria for the performance comparison. In order to demonstrate the robust performance of FLCD, we then compare predicted protein complexes from *three* selected state-of-the-art protein complex prediction algorithms based on *two* golden standard protein complex datasets on *four* public yeast PPI networks. What’s more, we apply GO enrichment analysis to the entire set of detected complexes by all the competing algorithms. At the end, we illustrate differences between protein complexes predicted by all competing algorithms corresponding to specific reference complexes to further demonstrate the superiority of our FLCD.

### Algorithms, data, and evaluation metrics

#### Algorithms

We compare our FLCD algorithm with other three state-of-the-art overlapping complex prediction algorithms, which are ClusterONE [6], LinkComm [12], and SR-MCL [9]. The JAVA implementation of ClusterONE does not require any tuning parameters. For LinkComm, we set the tuning parameter *t* (the threshold to cut the dendrogram for hierarchical clustering) to 0.2 that achieves the best performance empirically in our experiments. For SR-MCL, we set the inflation parameter *I*=3 and other parameters to their default settings since they yield the best results in our experiments. We set the only parameter *k* of our FLCD, the size of local neighbors based on personalized PageRank computation, to 20.

#### Data

We take four yeast PPI networks for performance evaluation: SceDIP, SceBG, SceIntAct, and SceMINT, extracted respectively from the Database of Interacting Proteins (DIP) [2], the Biological General Repository for Interaction Datasets (BioGRID) [3], the IntAct Molecular Interaction Database (IntAct) [4], and the Molecular INTeraction database (MINT) [5]. We note that we only consider protein-protein interactions by removing all genetic interactions from SceBG. We download the protein complex gold standards from the supplementary data in [6], which are obtained from the Saccharomyces Genome Database (SGD) [20] and the Munich Information Center for Protein Sequences (MIPS) [21] databases. For each PPI network, we remove reference protein complexes if their size smaller than 3 or half of the proteins of them are not in the network. The detailed information of four PPI networks and the gold standard reference complex datasets are provided in Table 1.

**Table 1:** The detailed information of four yeast PPI networks and the numbers of covered SGD and MIPS reference complexes

Network	#. proteins	#. interactions	SGD	MIPS
SceDIP	5136	22491	224	184
SceBG	6438	80577	234	189
SceIntAct	5453	54134	231	187
SceMINT	5414	27316	230	188

Due to the possible incompleteness of the reference protein complexes, we further examine the biological relevance of every predicted complex by GO enrichment analysis. We download the mappings of yeast genes and proteins to GO terms according to [20] (version 20150411).

#### Evaluation metrics for protein complex prediction

For the protein complex prediction, we assess the performance of all competing algorithms by a composite score consisting of three quality measures: F-measure [9, 14]; the geometric accuracy (Acc) score [14]; and the maximum matching ratio (MMR) [6]. For fair comparison, we remove predicted complexes of two or fewer proteins by all competing algorithms.

For a gold standard reference protein complex set *C*={*c*_1_,*c*_2_,…,*c*_*n*_} and a set of predicted complexes *S*={*s*_1_,*s*_2_,…,*s*_*m*_}, the F-measure is defined as the harmonic mean of precision and recall defined as follows:

1 $$ \textup{precision} = \frac{|N_{cs}|}{|S|}; \ \ \ \ \textup{recall} = \frac{|N_{cp}|}{|C|},  $$

in which *N*_*cs*_={*s*_*i*_∈*S*|*N* *A*(*c*_*j*_,*s*_*i*_)≥0.25,∃*c*_*j*_∈*C*} is the set of the complexes that match to one or more reference protein complexes; |*N*_*cs*_| is the size of the set *N*_*cs*_. *N*_*cp*_={*c*_*i*_∈*C*|*N* *A*(*c*_*i*_,*s*_*j*_)≥0.25,∃*s*_*j*_∈*S*} is a set of reference protein complexes that are matched by predicted complexes. We consider a reference protein complex *c*_*j*_ is matched by a predicted complex *s*_*j*_ if *N* *A*(*c*_*i*_,*s*_*j*_)≥0.25 [9, 22], where $NA(c_{i}, s_{j})=\frac {|c_{i} \cap s_{j}|^{2}}{|c_{i}| \times |s_{j}|}$ is called neighborhood affinity. Finally, the F-measure is

2 $$ {\textup{F-measure} = 2\times \frac{\textup{precision}*\textup{recall}}{\textup{precision}+\textup{recall}}}.  $$

The geometric accuracy (Acc) score is the geometric mean of two other measures — the cluster-wise sensitivity (Sn) and cluster-wise positive predictive value (PPV) [6]. Given *m* predicted and *n* reference complexes, let *t*_*ij*_ denote the number of proteins that exist in both predicted complex *s*_*i*_ and reference complex *c*_*j*_, and *w*_*j*_ represent the number of proteins in reference complex *c*_*j*_. Then Sn and PPV can be computed as

3 $$ \textup{Sn} = \frac{\sum_{j=1}^{n} \underset{i=1,\ldots, m}{max}t_{ij}}{\sum_{j=1}^{n} w_{j}}; \ \ \ \ \textup{PPV} = \frac{\sum_{i=1}^{m} \underset{j=1,\ldots, n}{max}t_{ij}}{\sum_{i=1}^{m}\sum_{j=1}^{n}t_{ij}}.  $$

The Acc score provides a balanced measure of Sn and PPV: $\textup {Acc}=\sqrt {\textup {Sn} \times \textup {PPV}}$.

The maximum matching ratio (MMR) is the ratio of the weight of maximum weight matching to the size of the reference set.

#### GO enrichment analysis

Suppose that a given PPI network has *N* proteins with *M* proteins annotated with one GO term and the predicted complex has *n* proteins with *m* proteins annotated with the same GO term. The *p*-value of the complex enriched with that GO term can be calculated as similarly done in [23]:

4 $$ {p}\text{-value} = \sum_{i=m}^{n} \frac{\binom{m}{i}\binom{N-M}{N-i}}{\binom{N}{n}}.  $$

We choose the lowest *p*-value of all its enriched GO terms for a predicted complex as its final *p*-value. A GO term is statistically significantly enriched when the *p*-value of any complex corresponding to this GO term is lower than 1*e*−3.

### Comparison on protein complex prediction

We apply all competing algorithms to search for potential protein complexes in four yeast PPI networks and compare them in terms of the composite score, consisting of F-measure, Acc score and MMR based on both the SGD and MIPS reference protein complex datasets.

We note that the different sizes and different numbers of detected complexes would affect the scores for the metrics that we have employed. However, in the context of complex prediction, there is no universal gold-standard metric. Hence, we apply three aforementioned metrics that have been commonly adopted in many other related works [6, 9]. We also note that the average sizes of the complexes generated by FLCD in our experiments are from 6 to 8 for four networks under study. The average complex sizes are indeed comparable to the average sizes of detected complexes by other algorithms. For example, the average sizes of complexes produced by LinkCommunity are from 5 to 6; The average sizes of complexes produced by ClusterONE are from 7 to 9; The average sizes of complexes produced by SR-MCL are from 8 to 10. Furthermore, the total numbers of predicted complexes yielded by FLCD, LinkCommunity and SR-MCL are much larger than that of ClusterONE. The reason is that the post-processing procedure of ClusterONE filters out complexes with lower scores but FLCD and LinkCommunity output all complexes without filtering.

**Fig. 1:**
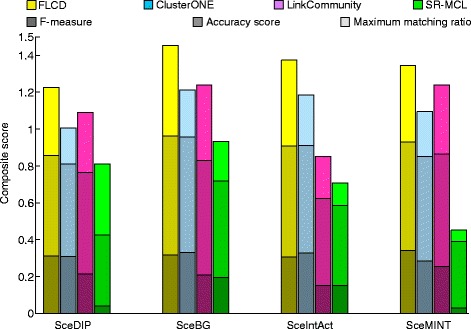
Comparison of all competing algorithms by SGD reference dataset in terms of the composite scores. *Shades of the same color*indicate different evaluating scores. Each bar height reflects the value of the composite score

**Fig. 2:**
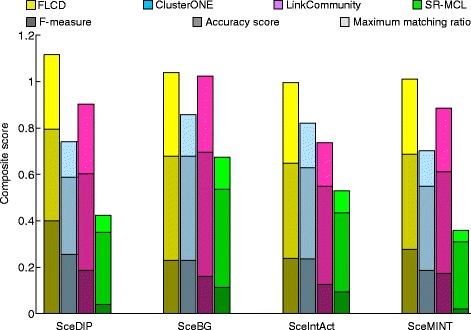
Comparison of all competing algorithms by MIPS reference dataset in terms of the composite scores. *Shades of the same color* indicate different evaluating scores. Each bar height reflects the value of the composite score

As shown in Figs. 1 and 2, FLCD clearly outperforms other state-of-the-art algorithms for all four networks on both SGD and MIPS reference datasets. Therefore, the complexes detected by FLCD have the best correspondence with the reference datasets. The detailed evaluation scores in Figs. 1 and 2 are displayed in Tables 2 and 3, respectively.

**Table 2:** Comparison of protein complex prediction by SGD reference dataset

Network	Method	# complex	#. matched	coverage	Recall	Precision	F-measure	Sn	PPV	Acc	MMR
SceDIP	FLCD	2134	**152**	3921	0.6786	0.2020	**0.3113**	0.5964	0.5003	0.5462	**0.3685**
	CONE	380	86	1503	0.3839	0.2579	0.3085	0.4082	0.6203	0.5032	0.1950
	LinkC	1839	137	3735	0.6116	0.1289	0.2130	0.6290	0.4820	**0.5506**	0.3276
	SR-MCL	3216	44	4678	0.2228	0.0221	0.0412	0.5120	0.2893	0.3489	0.0708
SceBG	FLCD	4027	**183**	5836	0.7821	0.2000	0.3181	0.7363	0.5621	**0.6433**	**0.4920**
	CONE	522	122	2735	0.5214	0.2433	**0.3318**	0.6488	0.6035	0.6257	0.2542
	LinkC	5382	164	6076	0.7008	0.1217	0.2072	0.8880	0.4373	0.6231	0.4100
	SR-MCL	1862	108	5889	0.4615	0.1245	0.1961	0.8999	0.3034	0.5225	0.2151
SceIntAct	FLCD	3394	**172**	4678	0.7446	0.1933	0.3069	0.6699	0.5391	**0.6009**	**0.4661**
	CONE	496	117	1994	0.5065	0.2419	**0.3275**	0.5742	0.5944	0.5842	0.2742
	LinkC	1297	93	5290	0.4026	0.0941	0.1525	0.9223	0.2393	0.4698	0.2285
	SR-MCL	1079	68	5342	0.2294	0.0437	0.1517	0.7784	0.2402	0.4341	0.1213
SceMINT	FLCD	2483	**157**	4210	0.6826	0.2280	**0.3418**	0.6524	0.5284	0.5871	**0.4163**
	CONE	513	110	2335	0.4783	0.2027	0.2848	0.5370	0.5954	0.5654	0.2442
	LinkC	2201	144	4068	0.6261	0.1595	0.2542	0.6757	0.5540	**0.6119**	0.3743
	SR-MCL	3698	33	4976	0.1435	0.0169	0.0302	0.5013	0.2597	0.3608	0.0609

CONE and LinkC are short for ClusterONE and LinkComm, respectively

Bold values denote the best scores corresponding to specific criteria

**Table 3:** Comparison of protein complex prediction by MIPS reference dataset

Network	Method	# complex	#. matched	Coverage	Recall	Precision	F-measure	Sn	PPV	Acc	MMR
SceDIP	FLCD	2134	**120**	3921	0.6522	0.1603	**0.2573**	0.4001	0.3901	0.3951	**0.3206**
	CONE	380	74	1503	0.4022	0.1868	0.2551	0.2749	0.4015	0.3322	0.1533
	LinkC	1839	109	3735	0.5924	0.1104	0.1862	0.4775	0.3646	**0.4173**	0.2993
	SR-MCL	2851	41	4687	0.1964	0.0230	0.0402	0.4592	0.2104	0.3108	0.0726
SceBG	FLCD	4027	**124**	5836	0.6561	0.1393	**0.2298**	0.4643	0.4315	0.4476	**0.3611**
	CONE	522	86	2735	0.4450	0.1533	0.2293	0.4537	0.4452	0.4494	0.1795
	LinkC	5382	109	6076	0.6349	0.0918	0.1604	0.8179	0.3504	**0.5354**	0.3285
	SR-MCL	1862	65	5889	0.3439	0.0673	0.1126	0.7360	0.2436	0.4234	0.1384
SceIntAct	FLCD	3394	**120**	4678	0.6417	0.1452	**0.2368**	0.4183	0.4034	0.4108	**0.3482**
	CONE	496	79	1994	0.4225	0.1633	0.2356	0.3587	0.4296	0.3925	0.1927
	LinkC	1297	80	5290	0.4278	0.0732	0.1251	0.9028	0.1986	**0.4234**	0.1886
	SR-MCL	1079	45	5342	0.1337	0.0190	0.0941	0.6246	0.1850	0.3399	0.0960
SceMINT	FLCD	2483	**111**	4210	0.5904	0.1800	**0.2759**	0.4147	0.4086	0.4116	**0.3231**
	CONE	513	67	2335	0.3564	0.1267	0.1869	0.3274	0.4017	0.3626	0.1519
	LinkC	2201	100	4068	0.5319	0.1040	0.1740	0.4744	0.4038	**0.4377**	0.2744
	SR-MCL	3698	24	4976	0.1277	0.0112	0.0205	0.4192	0.1999	0.2894	0.0481

CONE and LinkC are short for ClusterONE and LinkComm, respectively

Bold values denote the best scores corresponding to specific criteria

When we take SGD reference dataset as our gold standard protein complexes, from Table 2, we find that FLCD consistently achieves the best MMR scores among all competing algorithms because FLCD is the only algorithm that can capture the desired network structure of protein complexes. In the table, we also compare F-measure and the precision and recall scores that are used to compute F-measure. We observe that for all four PPI networks, FLCD predicts the largest number of matched reference protein complexes, and therefore FLCD attains the best recall scores for all PPI networks. With respect to the precision score, FLCD is the best for SceMINT but ClusterONE performs the best for the rest. However, since the post-processing step in ClusterONE only keeps the dense complexes, ClusterONE has low coverage. Based on the precision and recall scores, we find that FLCD attains the best F-measures for SceDIP and SceMINT PPI networks and ClusterONE obtains the best scores for SceBG and SceIntAct PPI networks. In addition to MMR and F-measure, we show comparison on the cluster-wise sensitivity (Sn), the cluster-wise positive predictive value (PPV) and the Acc score. We notice that FLCD has the best Acc scores for SceBG and SceIntAct. LinkComm obtains the best Acc scores for SceDIP and SceMINT, since LinkComm detects several large-size and many small-size complexes, which favors both the Sn and PPV scores [6]. We also compare the coverage of the competing algorithms and notice that SR-MCL has the largest coverage and FLCD has competitive coverage to SR-MCL. Here, the coverage is defined as the number of proteins covered by all predicted complexes, which is typically used to evaluate whether complex prediction algorithms can help comprehensively predict functionalities for all the proteins in a given network.

For MIPS reference dataset, we notice the similar trend for the evaluation scores in Table 3. FLCD finds the largest number of matched reference complexes in MIPS and attains the best recall scores, F-measures and MMR scores for all four PPI networks. The Acc scores of FLCD are competitive to LinkComm, which achieves the best Acc scores for all four yeast PPI networks. FLCD covers the competitive number of proteins to SR-MCL, which covers the largest number of proteins in all four yeast PPI networks. However, by the overall performance, which is represented by the composite score, FLCD is superior to other competing algorithms as shown in Fig. 2.

In summary, considering the composite score based on three metrics, our FLCD outperforms the other algorithms. To further validate all competing algorithms, we perform GO enrichment analysis in the next section to see whether all predicted complexes by different algorithms have significant biological meaning.

### Comparison on GO enrichment analysis

We perform GO enrichment analysis for all protein complexes predicted by the competing algorithms and report the percentages of the predicted protein complexes that are significantly enriched with at least one GO term and the total number of GO terms that are enriched in the predicted complexes in Table 4. We find that our FLCD achieves the best percentages of the enriched predicted protein complexes in SceDIP and SceIntAct PPI networks. ClusterONE obtains the best percentages for SceBG and SceMINT PPI networks but with the smaller number of GO terms enriched in the detected complexes because ClusterONE may remove meaningful functional modules in its post-processing step. Furthermore, the protein complexes detected by FLCD are significantly associated with the largest number of GO terms over all competing algorithms on all four PPI networks.

**Table 4:** Comparison by GO enrichment analysis

Network	Method	# complex	% enriched	# GO							
SceDIP	FLCD	2134	**72.2**	**1442**							
	CONE	380	71.8	852							
	LinkC	1839	67.4	1273							
	SR-MCL	2851	23.5	957							
SceBG	FLCD	4027	72.4	**1800**							
	CONE	522	**77.4**	1282							
	LinkC	5382	39.8	1554							
	SR-MCL	1862	56.4	1702							
SceIntAct	FLCD	3394	62.4	**1414**							
	CONE	496	**65.6**	1031							
	LinkC	1297	46.5	1129							
	SR-MCL	1079	44.7	888							
SceMINT	FLCD	2483	**62.3**	**1416**							
	CONE	513	59.4	954							
	LinkC	2201	32.1	1123							
	SR-MCL	3698	19.7	856							

“% enriched” presents the percentage of complexes that are enriched with at least one GO term. “# GO” denotes the number of enriched GO terms

Bold values denote the best scores corresponding to specific criteria

**Fig. 3:**
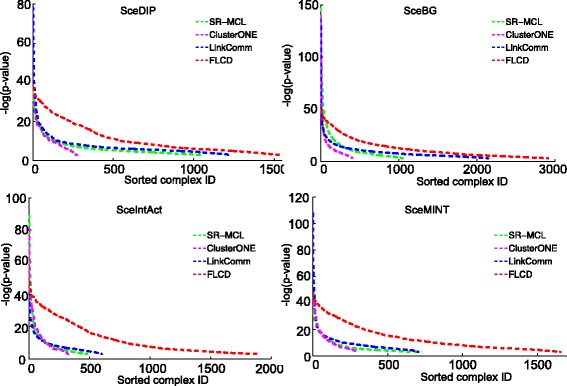
Statistical significance of the predicted complexes of all competing algorithms

To further examine the statistical significance of the complexes detected by the competing algorithms, we compare the *p*-values of the complexes under GO terms of biological process, molecular function, and cellular component domains. We use the lowest *p*-value for each predicted complex and show the comparison of the statistical significance of the complexes detected by all competing algorithms in Fig. 3. The y-axis of Fig. 3 represents the negative log-*p*-values while the x-axis is the ordered list of the complexes detected by all competing algorithms in terms of their negative log-*p*-values. Since complexes with significant biological relevance have lower *p*-values, higher values in Fig. 3 represent the higher quality of the detected complexes. As shown in Fig. 3, for all four yeast PPI networks, in addition to the fact that FLCD detects significantly more GO-enriched complexes, FLCD clearly outperforms other competing algorithms because the curves of FLCD are consistently on top of the others. The outperformance of FLCD further demonstrates that network structure that has dense internal connectivity and sparse external connectivity can better depict complexes of biological significance and FLCD provides an effective way to predict complexes with the desired network structure through explicitly taking care of internal and external connectivity of potential subnetworks.

### Examples of predicted complexes

**Fig. 4:**
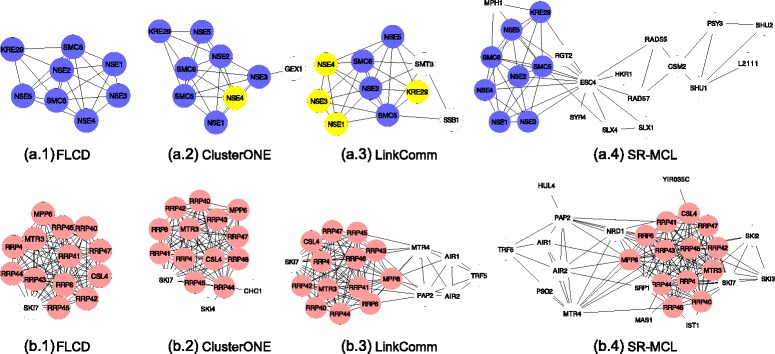
Illustrations of predicted complexes in SceBG network. ***a.1*** to ***a.4*** are Smc5-Smc6 complexes predicted by FLCD, ClusterONE, LinkComm, and SR-MCL, respectively. *Nodes in blue* are proteins in the reference Smc5-Smc6 complex and nodes in white are proteins outside the reference Smc5-Smc6 complex. *Nodes in yellow* are proteins failed to be detected by the corresponding algorithms. ***b.1*** to ***b.4*** are RNase complexes predicted by FLCD, ClusterONE, LinkComm, and SR-MCL, respectively. *Nodes in red* are proteins in the reference RNase complex and *nodes in white* are proteins outside the reference RNase complex

We further show the differences between the competing algorithms by illustrating the predicted complexes corresponding to two specific reference protein complexes. The first reference protein complex is the Smc5-Smc6 complex. In Fig. 4, the Smc5-Smc6 complexes predicted by FLCD, ClusterONE, LinkComm, and SR-MCL are displayed from (a.1) to (a.4), respectively. We notice that FLCD successfully identifies the Smc5-Smc6 complex as shown in Fig. 4(a.1). ClusterONE fails to detect the protein annotated as NSE4, probably due to the inaccuracy of the greedy algorithm used in ClusterONE. Also, we find that the protein annotated as GEX1 only interacts with the protein NSE3 but it is falsely added to the Smc5-Smc6 complex by ClusterONE. Because ClusterONE focuses on the separability of a complex but does not directly consider the internal density of the complex, it may mistakenly add proteins with small degrees into the final result. The complex in Fig. 4(a.3) predicted by LinkComm contains false positives and false negatives since the similarities between interactions used in LinkComm can not describe the topological structure of protein complexes. In Fig 4(a.4), we find out that the Smc5-Smc6 complex predicted by SR-MCL consists of many false positives. However, it is hard to explain the performance of SR-MCL on predicting the Smc5-Smc6 complex due to the unclear working mechanism of SR-MCL.

Similarly, we show the predicted RNase complexes by all competing algorithms in Fig. 4 from (b.1) to (b.4). In (b.1), we observe that FLCD detects all proteins in the reference RNase complex but mistakenly includes the protein SKI7 due to the existence of false positive interactions between SKI7 and proteins in RNase complex. In addition to SKI7, the predicted complex by ClusterONE (shown in Fig. 4(b.2)) contains two false positive proteins with very small degrees due to the ignorance of the internal density. Because LinkComm does not explicitly characterize the separability of the complexes, it also recruits some false positive proteins as clearly shown in Fig. 4(b.3). For the complex obtained by SR-MCL, we note that it has lots of false positive proteins and the topological property of the predicted complex is not clear.

## Conclusions

We propose an algorithm FLCD to predict protein complexes in protein-protein interaction networks. FLCD can better characterize the topological structure of a protein complex, which is densely connected inside and well separated from the rest of the networks. We compare FLCD with other three state-of-the-art algorithms on protein complex prediction. The comparison results show that FLCD achieves superior performances. Furthermore, GO enrichment analysis of the results of the competing algorithms demonstrates that FLCD finds more biologically meaningful complexes, within which proteins tend to be in the same cellular components and have similar functions and/or participate in the same biological processes.

## Methods

### Terminologies and definitions

Let an undirected graph *G*=(*V*,*E*) represent a PPI network, where *V* denotes the set of proteins in *G* and *E* is the interaction set. *A* is the adjacency matrix of *G* with *A*_*ij*_=*A*_*ji*_ and *A*_*ij*_=1 denoting node *i* interacts with node *j* and *A*_*ij*_=0 otherwise. The degree matrix *D* of *G* is a diagonal matrix with *D*_*ii*_=*d*_*i*_, where $d_{i} = \sum _{j} A_{ij}$ is the number of interactions connecting to protein *i*.

For a set *S* of proteins, the conductance of *S* in *G* is defined as [17]

5 $$ \phi(S) = \frac{|E(S, \bar{S})|}{\textup{min}\left \{ vol(S), vol(\bar{S}) \right \} }, \quad S \cup \bar{S} = V,  $$

where $E(S, \bar {S})$ denotes the edge cut, the set of edges between the set *S* and its complement set $\bar {S}$, |·| denotes the set size, and $vol(T) = \sum _{i\in T} d_{i}$ is the number of all incident interactions of the set *T*. Here we make a mild assumption that *v* *o* *l*(*S*)≪*v* *o* *l*(*V*) for a small protein complex *S* in the large-scale PPI network *G*, which means $vol(S) = \textup {min}\left \{ vol(S), vol(\bar {S}) \right \}$. Hence, we have

6 $$ \phi(S) = \frac{|E(S, \bar{S})|}{vol(S)} = \frac{\sum_{i} \left (D^{S}_{ii} - \sum_{j} A^{S}_{ij} \right)}{\sum_{i} D^{S}_{ii}},  $$

where *A* ^*S*^ is the adjacency matrix of the induced subnetwork with respect to set *S* and *D* ^*S*^ is the degree matrix for the nodes in *S*, where $D^{S}_{ii} = \sum _{j} A_{ij} = d_{i}$ for *i*∈*S*. For the same set *S*, the density of *S* is defined as [18]

7 $$ \mathcal{D}(S) = \frac{|E(S, S)|}{|S|}=\frac{1}{2}\frac{\sum_{ij}A^{S}_{ij} }{\sum_{i} \mathbf{1}_{i\in S}},  $$

where **1**_*i*∈*S*_ is the indicator function depending on whether *i*∈*S*.

### Motivation

**Fig. 5:**
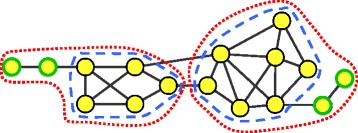
A motivating example for FLCD. *Red dotted lines* mark the complexes detected based on conductance minimization. *Blue dashed lines* mark the complexes predicted by our FLCD algorithm. Nodes with *green border lines* are removed by FLCD due to the lack of dense interactions

FLCD is motivated by conductance minimization to identify well separated subnetworks in a given network. However, FLCD can overcome the problem of conductance minimization, which pays no attention to the internal connectivity within subnetworks as potential protein complexes. Figure 5 shows a motivating example: We can find two complexes enclosed in the red dotted lines in the network based on conductance minimization. The conductances of the complexes within red dotted lines are $\frac {2}{11}$ and $\frac {2}{17}$ and the conductances of complexes within blue dashed lines are $\frac {3}{10}$ and $\frac {3}{16}$. Obviously, the conductances of the complexes within red dotted lines are lower than the complexes within blue dashed lines, indicating that the complexes within red dotted lines are topologically more separable than the complexes within blue dashed lines. However, the complexes within the blue dashed lines are more likely to be the desired complexes since the nodes with green border lines can not be confidently grouped into potential protein complexes due to their low degrees.

FLCD explicitly considers both the separability and internal edge density of complexes in two steps respectively. At the first step, it takes care of the separability of complexes by ensuring low conductance to hope for the complexes to have unique biological functions. At the second step, FLCD preserves the densely connected parts of the complexes identified in the first step. Because PPI networks are noisy and typically sparse, instead of finding cliques, we use the definition of internal density in (7) to search for dense subnetworks as final predicted complexes.

### Searching for a low-conductance set $\boldsymbol {H^{*}_{v}}$

Given a starting protein *v*, our goal is to find a protein set $H^{*}_{v}$ with low conductance including *v*. We first apply the algorithm proposed in [17] to find a potential set *H* with low conductance, then the minimum-conductance set $H^{*}_{v}$ in *H* is identified through solving a mixed integer programming (MIP) problem exactly.

Following [17], a low-conductance set including *v* can be efficiently approximated via the personalized PageRank vector of *v*. The personalized PageRank vector *p*(*α*,*v*) of *v* on *G* is the stationary distribution of the random walk on *G*, in which at every step, the random walker has the probability of *α* to restart the random walk at *v* and otherwise performs a lazy random walk. Mathematically, *p*(*α*,*v*) is the unique solution to

8 $$ p(\alpha, v) = \alpha e_{v} + (1-\alpha)p(\alpha, v) W,  $$

where *α*∈(0,1] is the “teleportation” constant, *e*_*v*_ is the indicator vector of *v* and $W=\frac {1}{2}(I + D^{-1}A)$ is the underlying probability transition matrix of the lazy random walk. We apply the local algorithm in [17] to efficiently approximate $\hat {p} \approx p(\alpha, v)$. Then we sort the nodes based on $\hat {p}$ and attain an ordered set $\mathcal {H} = \{ v_{1}, v_{2}, \ldots, v_{n} \}$, whose elements satisfy $\hat {p}(v_{i}) > \hat {p}(v_{i+1})$. Inspired by PageRank-Nibble [17] that sweeps the ordered set $\mathcal {H}$ to get the low-conductance set, we propose to find the minimum low-conductance set within a subnetwork of size *k*, which consists of the top *k* elements in $\mathcal {H}$, by solving a MIP problem. We take the top *k* elements out of $\mathcal {H}$, which are more likely to comprise a low-conductance set with *v*, and put them in *H*. The minimum-conductance set $H^{*}_{v}$ in *H* can be derived by solving the following optimization problem based on (6):

9 $$ \begin{aligned} \textup{min:} & \ \frac{x^{T}\left(D^{H}-A^{H}\right)x}{x^{T}d^{H}}\\ s.t. & \ {x_{v} = 1}, \ x_{i} \in \{0,1\}, \end{aligned}  $$

where *x* is a binary vector with *x*_*i*_=1 indicating that node *i* in *H* is assigned into $H^{*}_{v}$ and *x*_*i*_=0 otherwise; and *d* ^*H*^ is a vector containing the degrees of every node in *H*. We force node *v* to be in the low-conductance set by setting *x*_*v*_=1. By algebraic manipulations, (9) can be transformed into the following equivalent formulation:

10 $$ \begin{aligned} \textup{min:}& \ \ z\\ s.t. & \ \ z\sum_{i} x_{i}d^{H}_{i} - \sum_{i} \sum_{j} \left(D^{H}_{ij} - A^{H}_{ij}\right)x_{i}x_{j} \geq 0, \\ & \ \ {x_{v} = 1}, \ x_{i} \in \{0,1\}. \end{aligned}  $$

After using standard techniques [24] to linearize *z* *x*_*i*_ and *x*_*i*_ *x*_*j*_, the optimization problem can be solved by any MIP solver, such as Gurobi [25]. Because the size of |*H*|=*k* is much smaller than |*V*|=*n* and we only focus on identifying one low-conductance set, we can efficiently obtain the minimum-conductance set $H^{*}_{v}$ in *H* by solving (10) exactly.

If node *v* is in a connected component of size *k* ^′^ and we set *k*>*k* ^′^, then we might have a trivial solution that the low-conductance set is the connected component with conductance 0. To avoid this, we apply the following procedure. We check every derived low-conductance set of size *k* ^′^ to see whether it has exactly 0 conductance, which implies that it is a connected component with size *k* ^′^. If that is the case, we then set *k*=*k* ^′^−1, and re-solve the MIP to get a non-trivial solution.

### Conservation of the densest subnetwork $\boldsymbol {C_{v}^{*}}$ in $\boldsymbol {H^{*}_{v}}$

The induced subnetwork *G*_*v*_ with respect to the protein set $H^{*}_{v}$ is well separated from the rest of the network; however, there may exist nodes with low degrees in $H^{*}_{v}$. As illustrated in Fig. 5, to remove low-degree nodes (nodes with green border lines) as well as reserve densely connected subnetworks, we apply the definition of the internal density (7) to find the densest subnetwork in $H^{*}_{v}$. Because the problem size is small for such a local optimization problem, we can again take the full advantages of the power of MIP solvers. The node set $C_{v}^{*} \in H_{v}^{*}$ corresponding to the densest subnetwork can be identified based on (7) by deriving the exactly optimal solution to the following MIP problem:

11 $$ \begin{aligned} \textup{max:} & \ \ \frac{r^{T}A^{H_{v}^{*}}_{ij}r}{r^{T}\mathbf{1}} \\ s.t. & \ \ r_{i} \in \{0, 1\}, \end{aligned}  $$

where **1** is an all-one vector and *r* is the binary vector indicating the memberships of the nodes from $H^{*}_{v}$ in the densest subnetwork. This optimization problem explicitly searches for the subnetwork with the highest internal density and it can be transformed into the equivalent problem, as similarly done in (10):

12 $$ \begin{aligned} \textup{max:}& \ \ w\\ s.t. & \ \ w\sum_{i} r_{i} - \sum_{i} \sum_{j} A^{H_{v}^{*}}_{ij}r_{i}r_{j} \leq 0, \\ & \ \ r_{i} \in \{0,1\}, \end{aligned}  $$

which can also be cast into the MIP framework with the exactly optimal solution obtained by using standard MIP solvers after linearization [24].

### The FLCD algorithm

The step-by-step procedure of FLCD algorithm is given in Table 5. The FLCD algorithm screens every protein with degree higher than two. For each selected protein, the FLCD algorithm first searches for the minimum-conductance set around it and then finds the densest subnetwork in the minimum-conductance set, which is considered as a predicted complex. After screening every possible proteins, we remove the duplicated complexes and complexes with size smaller than three. There is only one parameter *k* for the FLCD algorithm, where *k* can be considered as the upper bound of the sizes of the desired protein complexes. Also, the MIP problems (10) and (12) are both NP hard. The actual computational complexity of solving these MIP problems depends on the problem size of these local problems determined by *k*. The smaller *k* is, the less time it takes the FLCD algorithm to search for subnetworks as potential protein complexes. Throughout the experiments in this paper, we set *k*=20.

**Table 5:** The FLCD algorithm

**Algorithm:** The FLCD Algorithm
**Input:** $\mathcal {S} = V$ and *k*=20.
**Output:** A set of predicted complexes *R*.
1 While ($\exists v \in \mathcal {S}$ and *d*_*v*_≥3)
2 Estimate $\hat {p} \approx p(\alpha, v)$.
3 Sort nodes in *V* based on $\hat {p}$ and collect the top *k* nodes in *H*_*v*_.
4 Finding the lowest-conductance set $H_{v}^{*}\in H_{v}$ based on (10).
5 Identifying the node set $C_{v}^{*}$ of the densest subnetwork in $H_{v}^{*}$ based on (12).
6 Considering $C_{v}^{*}$ as one predicted complex, let $R=\{R, C_{v}^{*}\}$ and$\mathcal {S} = \mathcal {S} - v$.
7 EndWhile
8 Remove duplicated complexes and complexes with size smaller than three in *R*.
